# Supplementary material for: Barriers and Facilitators for Implementing Music Interventions in Care Homes for People with Dementia and Depression: Process Evaluation Results of the Multinational Cluster-Randomized MIDDEL Trial
Source: Behav Sci (Basel). 2025 Jul 23;15(8):1004. doi: 10.3390/bs15081004 (PMC12383086; doi:10.3390/bs15081004)
Supplement: Supplementary file 1 [file behavsci-15-01004-s001.zip › Supplemental BS File S2. Staff survey.pdf]

Supplemental File S2.

Survey for care staff: baseline survey (T0)

The aim of this questionnaire is to gain insight into your expectations of the MIDDEL-project.

**Demographics**			
**Date** *(day month year)*		**Name CHU**	
**Age** *(in years)*		**Function within organization:**	☐ Registered nurse<br>☐ Enrolled nurse<br>☐ Personal care attendant<br>☐ Allied health professional<br>☐ Leisure staff<br>☐ Other
**Sex**	☐ Male ☐ Other<br>☐ Female	**Work experience** *(in years)*	
		**Are you familiar with music interventions in your organization?** *(check one box)*	☐ Yes, with music therapy<br>☐ Yes, with choir singing<br>☐ Yes, with both<br>☐ No
Has a contact person of the CHU already been appointed for the MIDDEL-project?		☐ Yes<br>☐ No ☐ I don't know ☐ Not applicable	

**Relevance and feasibility for the organization**	
Please select your level of agreement with each statement.<br>*1 = Strongly disagree, 2 = Disagree, 3 = Neither agree nor disagree, 4 = Agree, 5 = Strongly agree*	
**Recreational choir singing** will be relevant (meaningful, fitting, important) for the care home residents with dementia and depressive symptoms.	☐ 1 ☐ 2 ☐ 3 ☐ 4 ☐ 5
**Group music therapy** will be relevant for the care home residents with dementia and depressive symptoms.	☐ 1 ☐ 2 ☐ 3 ☐ 4 ☐ 5
**Recreational choir singing** will fit well into the day-to-day practice of the participating CHU(s).	☐ 1 ☐ 2 ☐ 3 ☐ 4 ☐ 5
**Group music therapy** will fit well into the day-to-day practice of the participating CHU(s).	☐ 1 ☐ 2 ☐ 3 ☐ 4 ☐ 5
I know which tasks I have to fulfill for the MIDDEL project.	☐ 1 ☐ 2 ☐ 3 ☐ 4 ☐ 5

**Barriers and facilitators: Care home organization and staff**		
**Step 1:** *Please indicate below for each factor or statement whether you expect this* ***will be applicable*** *within your organization during the MIDDEL-project.*		
**Statement**	**Yes**	**No**
The location is characterized by open communication structures and a "flat" hierarchy	☐	☐
Lack of support from care home management to implement the MIDDEL-project	☐	☐
Reorganizations: changes in responsibilities and care tasks	☐	☐
Participating in other studies or projects	☐	☐
In general, implementing an intervention on the CHU takes a lot of time (for example adopting a new health record system)	☐	☐
General resistance to change / trying out new things	☐	☐
Lack of care staff	☐	☐
High workload for care staff	☐	☐

**Barriers and facilitators: Care home organization and staff**					
**Step 2:** *Please indicate below for each factor (regardless of whether it is hindering or facilitating) to what extent you expect its presence may* ***influence the implementation*** *of the intervention.*					
**Statement**	**1: Not at all**	**2: To a small extent**	**3: To some extent**	**4: To a moderate extent**	**5: To a large extent**
The location is characterized by open communication structures and a "flat" hierarchy	☐	☐	☐	☐	☐
Lack of support from care home management to implement the MIDDEL-project	☐	☐	☐	☐	☐
Reorganizations: changes in responsibilities and care tasks	☐	☐	☐	☐	☐
Participating in other studies or projects	☐	☐	☐	☐	☐
In general, implementing an intervention on the CHU takes a lot of time (for example adopting a new health record system)	☐	☐	☐	☐	☐
General resistance to change / trying out new things	☐	☐	☐	☐	☐
Lack of care staff	☐	☐	☐	☐	☐
High workload for care staff	☐	☐	☐	☐	☐

**Barriers and facilitators: Music interventions**		
**Step 1:** *Please indicate below for each factor or statement whether you expect this* ***will be applicable*** *within your organization during the MIDDEL-project.*		
**Statement**	**Yes**	**No**
Changes in employees involved (e.g. team leaders, contact person, care staff, intervention providers)	☐	☐
Project planning is maintained by contact person, researchers and intervention providers	☐	☐
Clear communication between parties (contact person, care staff, intervention providers, researchers)	☐	☐
The people involved in the project facilitate and stimulate implementation of the project and interventions	☐	☐
Lack of motivation, commitment, enthusiasm amongst contact person, care staff, other co-workers, and intervention providers	☐	☐
Scarce resources (time, a space/room to conduct music sessions, music instruments)	☐	☐
There are musical activities/interventions in the care home	☐	☐
If yes: In general, music-based interventions/activities are well integrated in the care program of the care home	☐	☐
Difficulties/Discontinuity in performing the music interventions and assessments as intended due to COVID-19 pandemic	☐	☐

**Barriers and facilitators: Music interventions**					
**Step 2:** *Please indicate below for each factor (regardless of whether it is hindering or facilitating) to what extent you expect its presence may* ***influence the implementation*** *of the intervention.*					
**Statement**	**1: Not at all**	**2: To a small extent**	**3: To some extent**	**4: To a moderate extent**	**5: To a large extent**
Changes in employees involved (e.g. team leaders, contact person, care staff, intervention providers)	☐	☐	☐	☐	☐
Project planning is maintained by contact person, researchers and intervention providers	☐	☐	☐	☐	☐
Clear communication between parties (contact person, care staff, intervention providers, researchers)	☐	☐	☐	☐	☐
The people involved in the project facilitate and stimulate implementation of the project and interventions	☐	☐	☐	☐	☐

Barriers and facilitators: Music interventions					
Lack of motivation, commitment, enthusiasm amongst contact person, care staff, other co-workers, and intervention providers	☐	☐	☐	☐	☐
Scarce resources (time, a space/room to conduct music sessions, music instruments)	☐	☐	☐	☐	☐
There are musical activities/interventions in the care home	☐	☐	☐	☐	☐
If answered at step 1: In general, music-based interventions/activities are well integrated in the care program of the care home	☐	☐	☐	☐	☐
Difficulties/Discontinuity in performing the music interventions and assessments as intended due to COVID-19 pandemic	☐	☐	☐	☐	☐

**Implementation**					
Barriers and facilitators can make or break the implementation of a project. Please indicate below for both statements on a scale of 1 (very unlikely) to 5 (very likely) how likely it is that barriers to implement the music interventions will be solved or facilitators reinforced for the MIDDEL-project.					
**Statement**	**1: Very unlikely**	**2: Unlikely**	**3: Neutral**	**4: Likely**	**5: Very likely**
Anticipated facilitators will be reinforced.	☐	☐	☐	☐	☐
Anticipated barriers will be resolved.	☐	☐	☐	☐	☐

Survey for care staff: post-intervention survey (T6)

Demographics			
**Date** *(day month year)*		**Name of CHU**	
**Age** *(in years)*		**Function within organization:**	☐ Registered nurse<br>☐ Enrolled nurse<br>☐ Personal care attendant<br>☐ Allied health professional<br>☐ Leisure staff<br>☐ Other
**Sex**	☐ Male ☐ Other<br>☐ Female	**Work experience** *(in years)*	
		Were you involved in during the full length of the full length of the MIDDEL-project?	☐ Yes<br>☐ No, I got involved in (*month year*) _______________
**To what group(s) was/were the CHU(s) appointed?** *(check all that apply if multiple CHUs participated)*	☐ Group music therapy ☐ Both<br>☐ Recreational choir singing ☐ Care as usual		
Was there a contact person for the MIDDEL-project?	☐ Yes<br>☐ No ☐ I don't know ☐ Not applicable		
After the baseline assessment, how long did it take until the music intervention started?	☐ 2 weeks or less ☐ 2 – 4 weeks ☐ 4 – 6 weeks<br>☐ 6 – 8 weeks ☐ I don't know ☐ Not applicable		

Instruction	
Did you receive information (e.g. information letter or instruction session) explaining the MIDDEL-project?	☐ Yes ☐ No ☐ I don't know
Please select your level of agreement with each statement.<br>*1 = Strongly disagree, 2 = Disagree, 3 = Neither agree nor disagree, 4 = Agree, 5 = Strongly agree. NA = not applicable*	
I knew which tasks I had to fulfill for the MIDDEL project.	☐ 1 ☐ 2 ☐ 3 ☐ 4 ☐ 5 ☐ NA
I was satisfied with the information offered prior to the start of the project.	☐ 1 ☐ 2 ☐ 3 ☐ 4 ☐ 5 ☐ NA
The information, instruction, and guidance for the MIDDEL-project was sufficient.	☐ 1 ☐ 2 ☐ 3 ☐ 4 ☐ 5 ☐ NA
It was relevant to receive information prior the start of the MIDDEL-project.	☐ 1 ☐ 2 ☐ 3 ☐ 4 ☐ 5 ☐ NA

Relevance	
Please select your level of agreement with each statement.<br>*1 = Strongly disagree, 2 = Disagree, 3 = Neither agree nor disagree, 4 = Agree, 5 = Strongly agree. NA = not applicable*	
The music intervention was relevant (meaningful, fitting, important) for the care home residents with dementia and depressive symptoms.	☐ 1 ☐ 2 ☐ 3 ☐ 4 ☐ 5 ☐ NA
The music intervention was relevant **to reduce** depressive symptoms in care home residents with dementia.	☐ 1 ☐ 2 ☐ 3 ☐ 4 ☐ 5 ☐ NA
**Effect**	
Did you notice any effect(s) of the music intervention(s) on the CHU, in care staff, or in care home residents? *(select all that apply)*	☐ Yes, on the CHU ☐ No<br>☐ Yes, in care staff ☐ I don't know<br>☐ Yes, in residents ☐ Not applicable

**Feasibility**	
Please select your level of agreement with each statement.<br>*1 = Strongly disagree, 2 = Disagree, 3 = Neither agree nor disagree, 4 = Agree, 5 = Strongly agree*	
The music intervention fitted well into the day-to-day practice of the CHU.	☐ 1 ☐ 2 ☐ 3 ☐ 4 ☐ 5 ☐ NA
The music intervention turned out to be too complex to use.	☐ 1 ☐ 2 ☐ 3 ☐ 4 ☐ 5 ☐ NA
The music intervention was in line with how we are used to working.	☐ 1 ☐ 2 ☐ 3 ☐ 4 ☐ 5 ☐ NA
I would recommend the music intervention to other care homes.	☐ 1 ☐ 2 ☐ 3 ☐ 4 ☐ 5 ☐ NA
**Satisfaction**	
Please select your level of satisfaction:<br>*1 Not satisfied, 2 = Hardly satisfied, 3 = Somewhat satisfied, 4 = Satisfied, 5 = Completely satisfied*	
To what extent were you satisfied with the implementation of the music interventions?	☐ 1 ☐ 2 ☐ 3 ☐ 4 ☐ 5 ☐ NA

**Barriers and facilitators: Care home organization and staff**		
**Step 1:** *Please indicate below for each factor or statement whether this* **was applicable** *within your organization during the MIDDEL-project.*		
**Statement**	**Yes**	**No**
The location was characterized by open communication structures and a "flat" hierarchy	☐	☐
The location was characterized by stable, well-functioning teams	☐	☐
Lack of support from care home management to implement the MIDDEL project	☐	☐
Reorganizations: changes in responsibilities and care tasks	☐	☐
Participating in other studies or projects	☐	☐
General resistance to change / trying out new things	☐	☐
Lack of care staff	☐	☐
High workload for care staff	☐	☐

**Barriers and facilitators: Care home organization and staff**					
**Step 2:** *Please indicate below for each factor (regardless of whether it is hindering or facilitating) to what extent you feel like its presence* ***influenced the implementation*** *of the intervention.*					
**Statement**	**1: Not at all**	**2: To a small extent**	**3: To some extent**	**4: To a moderate extent**	**5: To a large extent**
The location was characterized by open communication structures and a "flat" hierarchy	☐	☐	☐	☐	☐
The location was characterized by stable, well-functioning teams	☐	☐	☐	☐	☐
Lack of support from care home management to implement the MIDDEL project	☐	☐	☐	☐	☐
Reorganizations: changes in responsibilities and care tasks	☐	☐	☐	☐	☐
Participating in other studies or projects	☐	☐	☐	☐	☐
General resistance to change / trying out new things	☐	☐	☐	☐	☐
Lack of care staff	☐	☐	☐	☐	☐
High workload for care staff	☐	☐	☐	☐	☐

**Barriers and facilitators: Music interventions**		
**Step 1:** *Please indicate below for each factor or statement whether this* **was applicable** *within your organization during the MIDDEL-project.*		
**Statement**	**Yes**	**No**
Changes in employees involved (team leaders, contact person, care staff, intervention providers)	☐	☐
Project planning is maintained by contact person, researchers and intervention providers	☐	☐
Clear communication between parties (contact person, care staff, intervention providers, researchers)	☐	☐
Negative attitude among those involved towards the appointed group for the MIDDEL-project (music intervention groups or control group)	☐	☐
The people involved in the project facilitate and stimulate implementation of the project and interventions	☐	☐
Lack of motivation, commitment, enthusiasm amongst contact person, care staff, other co-workers, and intervention providers	☐	☐
Scarce resources (time, a space/room to conduct music sessions, music instruments)	☐	☐
Implementing the music intervention(s) on the CHU took a lot of time	☐	☐
Difficulties/Discontinuity in performing the music interventions and assessments as intended due to COVID-19 pandemic	☐	☐

**Barriers and facilitators: Music interventions**					
**Step 2:** *Please indicate below for each factor (regardless of whether it is hindering or facilitating) to what extent you feel like its presence* ***influenced the implementation*** *of the intervention.*					
**Statement**	**1: Not at all**	**2: To a small extent**	**3: To some extent**	**4: To a moderate extent**	**5: To a large extent**
Changes in employees involved (team leaders, contact person, care staff, intervention providers)	☐	☐	☐	☐	☐
Project planning is maintained by contact person, researchers and intervention providers	☐	☐	☐	☐	☐
Clear communication between parties (contact person, care staff, intervention providers, researchers)	☐	☐	☐	☐	☐
Negative attitude among those involved towards the appointed group for the MIDDEL-project (music intervention groups or control group)	☐	☐	☐	☐	☐
The people involved in the project facilitate and stimulate implementation of the project and interventions	☐	☐	☐	☐	☐
Lack of motivation, commitment, enthusiasm amongst contact person, care staff, other co-workers, and intervention providers	☐	☐	☐	☐	☐
Scarce resources (time, a space/room to conduct music sessions, music instruments)	☐	☐	☐	☐	☐
Implementing the music intervention(s) on the CHU took a lot of time	☐	☐	☐	☐	☐
Difficulties/Discontinuity in performing the music interventions and assessments as intended due to COVID-19 pandemic	☐	☐	☐	☐	☐

**Implementation and sustainability**
Please indicate below for both statements on a scale of 1 (not at all) to 5 (to a large extent) to what extent barriers were solved or facilitators were reinforced for the MIDDEL-project.

Implementation and sustainability					
**Statement**	**1: Not at all**	**2: To a small extent**	**3: To some extent**	**4: To a moderate extent**	**5: To a large extent**
Expected/anticipated facilitators were reinforced.	☐	☐	☐	☐	☐
Expected/anticipated barriers were resolved.	☐	☐	☐	☐	☐
To what degree, in your opinion, was the music intervention implemented on the CHU?	☐	☐	☐	☐	☐
Will the music intervention(s) continue on the participating CHU(s) after this 6-month intervention period?	☐ Yes → _____ times a week<br>☐ No ☐ I don't know				

Survey for care staff: follow-up survey (T12)

**Demographics**			
**Date** *(day month year)*		**Name CHU**	
**Age** *(in years)*		**Function within organization:**	☐ Registered nurse<br>☐ Enrolled nurse<br>☐ Personal care attendant<br>☐ Allied health professional<br>☐ Leisure staff<br>☐ Other
**Sex**	☐ Male ☐ Other<br>☐ Female	**Work experience** *(in years)*	
		Were you involved in during the full length of the full length of the MIDDEL-project?	☐ Yes<br>☐ No, I got involved in (*month year*) ________________
**To what group(s) was/were the CHU(s) appointed?** *(check all that apply if multiple CHUs participated)*		☐ Group music therapy ☐ Both<br>☐ Recreational choir singing ☐ Care as usual	
Did the music intervention(s) continue on the participating CHU(s) after this 6-month intervention period?		☐ Yes → _____ times a week<br>☐ No ☐ I don't know	

**Implementation and sustainability**					
Please indicate below for both statements on a scale of 1 (not at all) to 5 (to a large extent) to what extent barriers were solved or facilitators were reinforced for the MIDDEL-project.					
**Statement**	**1: Not at all**	**2: To a small extent**	**3: To some extent**	**4: To a moderate extent**	**5: To a large extent**
Facilitators for continuation of the music intervention were reinforced.	☐	☐	☐	☐	☐
Barriers for continuation of the music intervention were resolved.	☐	☐	☐	☐	☐
To what degree, in your opinion, was the music intervention implemented on the CHU?	☐	☐	☐	☐	☐

**Relevance**	
Please select your level of agreement with each statement.<br>*1 = Strongly disagree, 2 = Disagree, 3 = Neither agree nor disagree, 4 = Agree, 5 = Strongly agree. NA = not applicable*	
The music intervention was relevant (meaningful, fitting, important) for the care home residents with dementia and depressive symptoms.	☐ 1 ☐ 2 ☐ 3 ☐ 4 ☐ 5 ☐ NA
The music intervention was relevant **to reduce** depressive symptoms in care home residents with dementia.	☐ 1 ☐ 2 ☐ 3 ☐ 4 ☐ 5 ☐ NA
**Effect**	
Did you notice any effect(s) of the music intervention(s) on the CHU, in care staff, or in care home residents? *(select all that apply)*	☐ Yes, on the CHU ☐ No<br>☐ Yes, in care staff ☐ I don't know<br>☐ Yes, in residents ☐ Not applicable

**Feasibility**	
Please select your level of agreement with each statement.<br>*1 = Strongly disagree, 2 = Disagree, 3 = Neither agree nor disagree, 4 = Agree, 5 = Strongly agree*	
The music intervention fitted well into the day-to-day practice of the CHU(s).	☐ 1 ☐ 2 ☐ 3 ☐ 4 ☐ 5 ☐ NA
The music intervention turned out to be too complex to use.	☐ 1 ☐ 2 ☐ 3 ☐ 4 ☐ 5 ☐ NA
The music intervention was in line with how we are used to working.	☐ 1 ☐ 2 ☐ 3 ☐ 4 ☐ 5 ☐ NA
I would recommend the music intervention to other care homes.	☐ 1 ☐ 2 ☐ 3 ☐ 4 ☐ 5 ☐ NA

**Barriers and facilitators: Care home organization and staff**		
**Step 1:** *Please indicate below for each factor or statement whether this* **was applicable** *within your organization during the MIDDEL-project.*		
**Statement**	**Yes**	**No**
The location was characterized by open communication structures and a "flat" hierarchy	☐	☐
The location was characterized by stable, well-functioning teams	☐	☐
Lack of support from care home management to implement the MIDDEL-project	☐	☐
Reorganizations: changes in responsibilities and care tasks	☐	☐
Participating in other studies or projects	☐	☐
General resistance to change / trying out new things	☐	☐
Lack of care staff	☐	☐
High workload for care staff	☐	☐

**Barriers and facilitators: Care home organization and staff**					
**Step 2:** *Please indicate below for each factor (regardless of whether it is hindering or facilitating) to what extent you feel like its presence* ***influenced the implementation*** *of the intervention.*					
**Statement**	**1: Not at all**	**2: To a small extent**	**3: To some extent**	**4: To a moderate extent**	**5: To a large extent**
The location was characterized by open communication structures and a "flat" hierarchy	☐	☐	☐	☐	☐
The location was characterized by stable, well-functioning teams	☐	☐	☐	☐	☐
Lack of support from care home management to implement the MIDDEL-project	☐	☐	☐	☐	☐
Reorganizations: changes in responsibilities and care tasks	☐	☐	☐	☐	☐
Participating in other studies or projects	☐	☐	☐	☐	☐
General resistance to change / trying out new things	☐	☐	☐	☐	☐
Lack of care staff	☐	☐	☐	☐	☐
High workload for care staff	☐	☐	☐	☐	☐

**Barriers and facilitators: Music interventions**		
**Step 1:** *Please indicate below for each factor or statement whether this* **was applicable** *within your organization during the MIDDEL-project.*		
**Statement**	**Yes**	**No**
Changes in employees involved (team leaders, contact person, care staff, intervention providers)	☐	☐
Project planning is maintained by contact person, researchers and intervention providers	☐	☐

**Barriers and facilitators: Music interventions**		
**Step 1:** *Please indicate below for each factor or statement whether this* ***was applicable*** *within your organization during the MIDDEL-project.*		
**Statement**	**Yes**	**No**
Clear communication between parties (contact person, care staff, intervention providers, researchers)	☐	☐
Negative attitude among those involved towards the appointed group for the MIDDEL-project (music intervention groups or control group)	☐	☐
The people involved in the project facilitate and stimulate implementation of the project and interventions	☐	☐
Lack of motivation, commitment, enthusiasm amongst contact person, care staff, other co-workers, and intervention providers	☐	☐
Scarce resources (time, a space/room to conduct music sessions, music instruments)	☐	☐
Preparing, executing and keeping the music intervention(s) running was a lot of work	☐	☐
Difficulties/Discontinuity in performing the music interventions and assessments as intended due to COVID-19 pandemic	☐	☐

**Barriers and facilitators: Music interventions**					
**Step 2:** *Please indicate below for each factor (regardless of whether it is hindering or facilitating) to what extent you feel like its presence* ***influenced the implementation*** *of the intervention.*					
**Statement**	**1: Not at all**	**2: To a small extent**	**3: To some extent**	**4: To a moderate extent**	**5: To a large extent**
Changes in employees involved (team leaders, contact person, care staff, intervention providers)	☐	☐	☐	☐	☐
Project planning is maintained by contact person, researchers and intervention providers	☐	☐	☐	☐	☐
Clear communication between parties (contact person, care staff, intervention providers, researchers)	☐	☐	☐	☐	☐
Negative attitude among those involved towards the appointed group for the MIDDEL-project (music intervention groups or control group)	☐	☐	☐	☐	☐
The people involved in the project facilitate and stimulate implementation of the project and interventions	☐	☐	☐	☐	☐
Lack of motivation, commitment, enthusiasm amongst contact person, care staff, other co-workers, and intervention providers	☐	☐	☐	☐	☐
Scarce resources (time, a space/room to conduct music sessions, music instruments)	☐	☐	☐	☐	☐
Preparing, executing and keeping the music intervention(s) running was a lot of work	☐	☐	☐	☐	☐
Difficulties/Discontinuity in performing the music interventions and assessments as intended due to COVID-19 pandemic	☐	☐	☐	☐	☐
