# Supplementary material for: Barriers and Facilitators for Implementing Music Interventions in Care Homes for People with Dementia and Depression: Process Evaluation Results of the Multinational Cluster-Randomized MIDDEL Trial
Source: Behav Sci (Basel). 2025 Jul 23;15(8):1004. doi: 10.3390/bs15081004 (PMC12383086; doi:10.3390/bs15081004)
Supplement: Supplementary file 1 [file behavsci-15-01004-s001.zip › Supplemental BS File S1. Explanatory note survey development.pdf]

Supplemental File S1.

Explanatory note to paragraph *2.4 Survey development*

The survey was adapted to be appropriate for the targeted stakeholder, meaning the survey had minor differences for each stakeholder: management, care staff, and interventionists. To illustrate, the baseline (T0) survey questions and statements were formulated in simple future tense (e.g., “Recreational choir singing will be relevant for the care home residents with dementia and depressive symptoms.” and “Please indicate below for each factor (regardless of whether it is hindering or facilitating) to what extent you expect its presence may influence the implementation of the intervention.”). Items of the post-intervention survey T6 were formulated in the past tense (e.g., “To what extent were you satisfied with the implementation of the MIDDEL-project?” and “Please indicate below for each factor or statement whether this was applicable within your organization during the MIDDEL-project”). A single statement, for which stakeholders indicated to what extent a factor was present, was worded differently per stakeholder. The statement for managers was “Lack of support from board of directors to implement the MIDDEL-project”, whereas the statement for care staff was “Lack of support from care home management to implement the MIDDEL project”.
